# A Novel Application for Low Frequency Electrochemical Impedance Spectroscopy as an Online Process Monitoring Tool for Viable Cell Concentrations

**DOI:** 10.3390/s16111900

**Published:** 2016-11-11

**Authors:** Christoph Slouka, David J. Wurm, Georg Brunauer, Andreas Welzl-Wachter, Oliver Spadiut, Jürgen Fleig, Christoph Herwig

**Affiliations:** 1 Research Division Biochemical Engineering, Institute of Chemical Engineering, Vienna University of Technology, Vienna 1060, Austria; david.wurm@tuwien.ac.at (D.J.W.); oliver.spadiut@tuwien.ac.at (O.S.); christoph.herwig@tuwien.ac.at (C.H.); 2 Institute for Energy Systems and Thermodynamics, Vienna University of Technology, Vienna 1060, Austria; georg.brunauer@tuwien.ac.at; 3 Research Division Electrochemistry, Institute of Chemical Technology and Analytics, Vienna University of Technology, Vienna 1060, Austria; andreas.welzl@tuwien.ac.at (A.W.-W.); juergen.fleig@tuwien.ac.at (J.F.); 4 Christian Doppler Laboratory for Mechanistic and Physiological Methods for Improved Bioprocesses, Institute of Chemical Engineering, Vienna University of Technology, Vienna 1060, Austria

**Keywords:** *Escherichia coli*, viable cell count, online biomass monitoring, impedance spectroscopy

## Abstract

New approaches in process monitoring during industrial fermentations are not only limited to classical pH, dO_2_ and offgas analysis, but use different in situ and online sensors based on different physical principles to determine biomass, product quality, lysis and far more. One of the very important approaches is the in situ accessibility of viable cell concentration (VCC). This knowledge provides increased efficiency in monitoring and controlling strategies during cultivations. Electrochemical impedance spectroscopy—EIS—is used to monitor biomass in a fermentation of *E. coli* BL21(DE3), producing a recombinant protein using a fed batch-based approach. Increases in the double layer capacitance (C_dl_), determined at frequencies below 1 kHz, are proportional to the increase of biomass in the batch and fed batch phase, monitored in offline and online modes for different cultivations. A good correlation of C_dl_ with cell density is found and in order to get an appropriate verification of this method, different state-of-the-art biomass measurements are performed and compared. Since measurements in this frequency range are largely determined by the double layer region between the electrode and media, rather minor interferences with process parameters (aeration, stirring) are to be expected. It is shown that impedance spectroscopy at low frequencies is a powerful tool for cultivation monitoring.

## 1. Introduction

Microbial cultivations play a key role in many different fields such as food, drug and bulk chemical production as well as in waste to value concepts [1]. Process monitoring such as pH, dO_2_, offgas analysis and biomass measurements are state of the art in today’s industrial cultivations to guarantee product quality and safety. Generally, in industrial processes, produced biomass estimation and closed loop control can be established through soft sensor applications [2]. However, these control systems are dependent on atline detection systems such as high performance liquid chromatography for metabolite measurements. Therefore, the accurate and reliable measurement of biomass [3,4] and especially of viable cell concentrations (VCC) during cultivations increases the accuracy of given input parameters and increases the efficiency of these process control tools.

VCC is measured using offline measurement principles including marker proteins or fluorescence probes, such as flow cytometry or confocal microscopy [5,6]. Online and inline approaches are rather scarce and are based on physical measurement principles. One principle generally applied is high frequency alternating current (AC) impedance spectroscopy with high field amplitudes used on the basis of the so called β-dispersion [7,8]. Cells with an integer cell membrane affect the relative permittivity between two electrodes and therefore this signal is used for the estimation of VCC. A detailed description of the measurement principles can be found in References [9,10,11,12].

The model organism for the application of AC measurements in the β-dispersion range is yeast, as it is a very important expression host for recombinant proteins [13,14,15]. Also, approaches towards more complex expression systems, such as filamentous fungi and chinese hamster ovary (CHO) cells, are already performed [16,17,18,19]. In general, these measurements show a strong dependence upon physical process parameters (such as aeration and stirring—causing gas bubbles, temperature shifts and pH gradients) and are furthermore highly affected by changes in the media composition during cultivation (f.e. sugar concentrations).

However, not only high frequency impedance spectroscopy in the ß-range can be used for the determination of biomass, but also changes of the double layer of cells with the electrode surface (detectable at low frequencies in the mHz range, α-dispersion) provide valuable information. Beside the cell type itself (cell wall/membrane compositions, size and shape), many physical parameters, especially in the media (pH, ion concentrations), can influence the potential distribution in the double layer [20,21]. Furthermore, the given method is capable of detecting even very small numbers of bacteria in soil, food and feces-polluted water using interdigitated microelectrode designs [22,23,24,25,26,27].

These studies were only performed at a very small scale with a low cell concentration. In general, a threshold in the measurement is present at a low cell concentration. Exceeding this limitation, very stable signals over time were achieved. Beside direct measurements in the broth, a modified electrode system in an interdigitated design can be used [28,29,30]. First approaches towards process monitoring were shown by Kim et al. [31], who worked with an inline sensor used in the lower frequency range between 40 Hz and 10 kHz for real-time monitoring of biomass. Kim et al. showed the feasibility for measuring changes in the double layer capacitance, but no analysis of the double layer capacitance (C_dl_) itself was performed; only discrete extracted values for distinct frequency values were used. Furthermore, this was done only in a batch cultivation approach and no online or offline analysis of cell physiology, which is essential for differentiation between different cell states, e.g., living/dead counting, were shown. This can lead to overestimation of the measured cell density and results in additional uncertainties during the measurement.

In this study, impedance measurements during a fed batch–based cultivation, as used in industrial-scale bioreactors, with *E. coli* BL21(DE3), producing a recombinant cytosolic protein, are presented. For calibration of the signal, offline and online measurements of the impedance are correlated to measured biomass. Different state-of-the-art methods are applied for determination of the corresponding total biomass—dry cell weight (DCW), OD_880_ inline and OD_600_ offline. Flow cytometry (FCM) in combination with different fluorescence dyes is used for cell physiology evaluation to account for changes in the viability during cultivation. With this knowledge, we are able to correlate the total biomass to the extracted double layer capacitance.

Within this study, a prototype online probe (flow-through cell) was designed and built. With this easy-to-rebuild probe we show an excellent correlation between double layer capacitance and viable cell concentration which allows online cell concentration monitoring with high accuracy over a very broad cell concentration (1 g/L to 40 g/L investigated in this study).

## 2. Materials and Methods

### 2.1. Expression Host and Cultivation

All cultivations were performed using an *E. coli* BL21(DE3) strain as expression host transformed to produce recombinant horseradish peroxidase (HRP) (pet39+/HRP) or a recombinant cytoplasmic antibody fragment. For the preculture 500 mL sterile DeLisa medium was inoculated from frozen stocks (1.5 mL, −80 °C) and incubated in a 2500 mL High-Yield shake flask for 20 h (230 rpm, 37 °C). Batch and fed batch cultivations were performed in a stainless steel Sartorius Biostat Cplus bioreactor (Sartorius, Göttingen, Germany) with 10 L working volume. A batch and fed-batch phase for biomass generation were followed by an induction phase using a mixed feed medium with glucose as primary carbon source and lactose as carbon source as well as inducer. Detailed information about the bioreactor setup and media composition can be found elsewhere [32].

### 2.2. Analytics

For DCW measurements 2 mL of the cultivation broth was centrifuged at 4500× *g*, subsequently washed with 0.9% NaCl solution and centrifuged again. After drying the cells at 105 °C for 48 h the pellet was evaluated gravimetrically. DCW measurements were performed in triplicates and the mean error for DCW was always 3%. Offline OD_600_ measurements were performed in duplicates in a UV/VIS photometer Genisys 20 (Thermo Scientific, Waltham, MA, USA).

For inline OD_880_ measurements a Dencytee total cell density measurement cell (Hamilton, Reno, NV, USA) was used. In general, at cell densities above 20 g/L (DCW) saturation effects were observed [33] (exceeding linear range of Lambert Beer’s law as already observed for offline OD_600_ measurements). Verification of cell viability was done by flow cytometric (FCM) measurements. After addition of DiBAC4 (bis-(1,3-dibutylbarbituricacid)trimethineoxonol) and Rh414 dye diluted cultivation broth was measured using a CyFlow Cube 8 flow cytometer (Sysmex-Partec, Bornbach, Germany). Rh 414 binds to the plasma membrane and visualizes all cells, while DiBAC is sensitive to plasma membrane potential and therefore distinction between viable and non-viable cells can be achieved. Detailed information on the viability assay can be found elsewhere [34]. Overall errors with this method were in the range of 0.5% to 1%. As less than 5% of dead cells were detected in all samples, DCW and VCC can be assumed to be equivalent.

## 3. Results

Within this study we developed a method to estimate viable cell concentration by measuring low frequency electrochemical impedance spectra during cultivation. Therefore, we (1) constructed a prototype probe and developed a method to link the impedance signal to the viable cell concentration. As the signals of the physical measurement probes are often dependent on changing process parameters and media composition; (2) we investigated the impact of changing the media background on the measurement during the cultivation. Finally; (3) we implemented the constructed impedance probe in the online mode to show the feasibility of this novel biomass sensor.

### 3.1. Construction of the Prototype Online Probe and Data Processing

**Figure 1:**
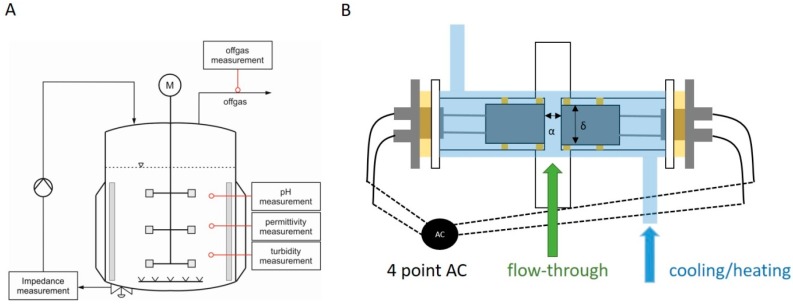
(**A**) Schematic drawing of the fermentation setup using the online impedance probe. A peristaltic pump establishes the flow through the probe; (**B**) Schematic drawing of the prototype online probe during fermentation. The stainless steel electrodes are sealed in borosilicate tubing (NW16KF) and a polymethymetacrylate mantle. An electrical connection is established using a four-point measuring method to decrease cable induction and setup interferences.

Before the construction of a prototype probe, samples were measured in the offline mode by pipetting the samples into a glass cuvette with incorporated electrodes for measuring the capacitance signal. Stainless steel electrodes were used as they are described in literature to give good signals for analyzing bacteria [27]. For online measurements, we constructed a thermostatically controlled flow cell, which was connected to the bioreactor by a peristaltic pump and automatically recorded the signal in regular time intervals. The flow cell and the experimental setup for online application are presented in Figure 1.

To facilitate rebuilding the online probe, commercially available standardized parts were used. The flow cell was made of a borosilicate glass cylinder using DN 16 glass cylinders with an adaptor connection. The continuous flow through the flow cell was maintained by connecting a peristaltic pump to the sampling port and pumping the cultivation broth continuously through the probe (exceeding 100 mL/min). The outer heating/cooling jacket was made of polymethymetacrylate cylinders and boards and allowed temperature control of the probe by connecting the double jacket to a heating and cooling thermostat alpha-RA (Lauda, Lauda-Königshofen, Germany). We chose the distance between the electrodes to be α = 1.4 cm (see Figure 1) to guarantee a stable and fast flow through the probe without pressure loss. The diameter of the circular electrodes was designed to be δ = 1.3 cm (see Figure 1) to fit into the DN16 glass cylinders. Electrical contact was established by soldering onto commercial available “Bayonet Neill Concelman” (BNC) cables.

Physical analysis of VCC in state-of-the-art capacitance probes, which rely on β-dispersion (10^7^ Hz–10^4^ Hz), showed high dependence on process parameters (e.g., stirring, temperature, pH, salt and substrate concentration, etc.) and the cultivation phase (exponential growth phase, starvation phase, etc.) [11,19]. We focused the measurement on a different physical phenomenon (α-dispersion), which yields valuable information mainly about biomass concentration. The so called α-dispersion effect, at frequencies below 10 kHz, which is most probably a result of the deformation of ionic species around the cell membranes, is used for these measurements. The dielectric response is therefore proportional to the ionic charge gathered around the membrane of adsorbed cells on the electrode [20,21]. Impedance measurements were recorded in the range of 10^6^ to 10^−1^ Hz with amplitudes between 100 and 500 mV using an Alpha-A High Resolution Dielectric Analyzer or a Pot/Gal measuring interface (Novocontrol, Montabaur, Germany). Since measurements in this frequency range are largely determined by the double layer region between the electrode and media, rather minor interferences with process parameters (aeration, stirring) were to be expected.

**Figure 2:**
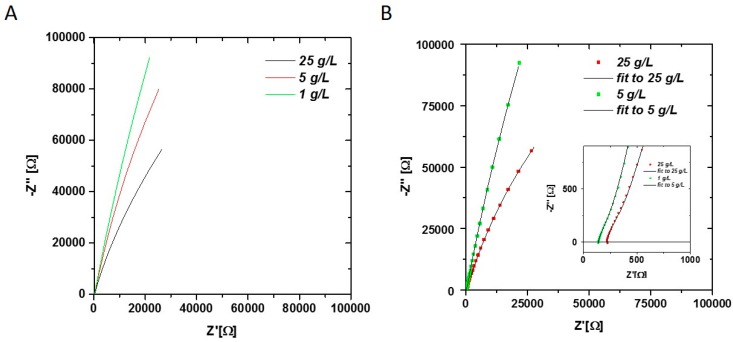
(**A**) Nyquist plot of *E. coli* samples at different concentrations (1–25 g/L) measured in offline mode. Obvious changes in the double layer are visible at low frequencies; (**B**) Nyquist plot of 1 g/L and 25 g/L sample with fit using equivalent circuit in Equation (1). Fitted parameters all three samples are given in Table 1. The Q value of the CPE_dl_ element is strongly dependent on the biomass concentration.

To show the feasibility of monitoring the biomass concentration by impedance spectroscopy, *E. coli* cells were measured at different concentrations (Figure 2).

An obvious increase of the capacitance (arc bending to the right) in the Nyquist plot is visible between high and low biomass concentrations (Figure 2A), which proves the applicability of the chosen method to differentiate between different cell concentrations.

To explain the shape of the Nyquist plot and also to obtain parameters which can be used to establish a correlation between the cell concentration and impedance signal, the following equivalent circuit, including the media contributions [25], was chosen to mechanistically describe the data (Equation (1)).

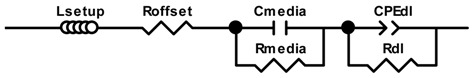
(1)

The circuit contains an inductance and a resistance term for setup correction (L_setup_ and R_offset_), a resistance-capacitance element for the media contributions (R_media_-C_media_) (high frequency shoulder visible in the Niquist plot—inlay Figure 2B) and a parallel connection of the resistance and constant phase element for the double layer contribution (R_dl_-CPE_dl_).

The impedance (Z) of a general resistance and a non-ideal capacitance R-CPE element (the connection of a resistor (R) and a constant phase element (CPE)) can be expressed by Equation (2).

(2) $$Z=\frac{1}{{\left(i\omega \right)}^{n}Q}$$

where ω is the arc frequency and i is the imaginary number; n and Q are parameters that need to be fitted and are used to calculate the sample capacitance (C) according to Equation (3) [35].

(3) $$C={\left(R^{1-n}Q\right)}^{1/n}$$

At high cell densities, R_dl_ can be fitted and reflects changes in the ionic composition of the double layer. At low cell concentrations, R_dl_ is not accessible due to a high overall fitting error (see Table 1) and leads to a high error of the calculated sample capacitance (C) according to Equation (3).

The data obtained from the impedance measurements of the offline biomass samples were fitted according to the proposed equivalent circuit shown in Equation (1) by complex non-linear least square fitting (CNLS) using the software ZView (Scribner, Southern Pines, NC, USA). The results for the fit (displayed in Figure 2B) and the corresponding parameters are given in Table 1.

The CPE-n value is a marker for the non-ideality of the corresponding capacitance. This value can be evaluated from the experimental data and Equation (1), and is mostly in an acceptable range between 0.8 to 0.9 (vs. 1.00 for an ideal capacitance) to warrant calculation of a reasonable C_DL_ value. Therefore, we used CPE_dl_-Q values instead of the calculated sample capacitance for establishing a correlation between cell concentration and impedance data to reduce the error and extend the applicability of this measurement method also to low cell concentrations, where R_dl_ has a high error. All fitted parameters give physically reasonable values and a low NRMSE between 0% and 10% (except for R_dl_ at low biomass concentrations) which indicates that the proposed model (Equation (1)) is valid. For all further analysis, CPE_dl_-Q was used to correlate the viable cell concentration to the impedance spectra.

### 3.2. Impact of Changing Media Background on Impedance Measurements

As already shown, the developed method is capable of measuring changes in the cell concentration as a function of the double layer capacitance. To apply this method also in the online mode, the impact of the media composition, which generally changes during a cultivation, on the measurement was investigated by measuring samples in the offline mode. Therefore, (1) a cultivation medium with different sugar concentrations; (2) centrifuged cultivation supernatants at different process times with different salt, sugar and host cell protein concentrations; (3) cultivation broth with cells at different process times; and (4) cells resuspended in the same matrix (cultivation medium) at different cell concentrations were analyzed.

For determination of the interferences of sugars with the measurement, cultivation media supplemented with up to 200 g/L of glucose, lactose and galactose were tested. Differences in the monosaccharide concentration seem to have no effect on the measured signal. Only at very high concentrations of lactose (200 g/L) minor changes (not shown) in the double layer capacitance were observed, but these high concentrations usually do not occur during *E. coli* cultivations.

To investigate the impact of produced metabolites, changing salt and sugar concentration samples were taken throughout a bioreactor cultivation which yielded up to 30 g/L DCW biomass.

**Figure 3:**
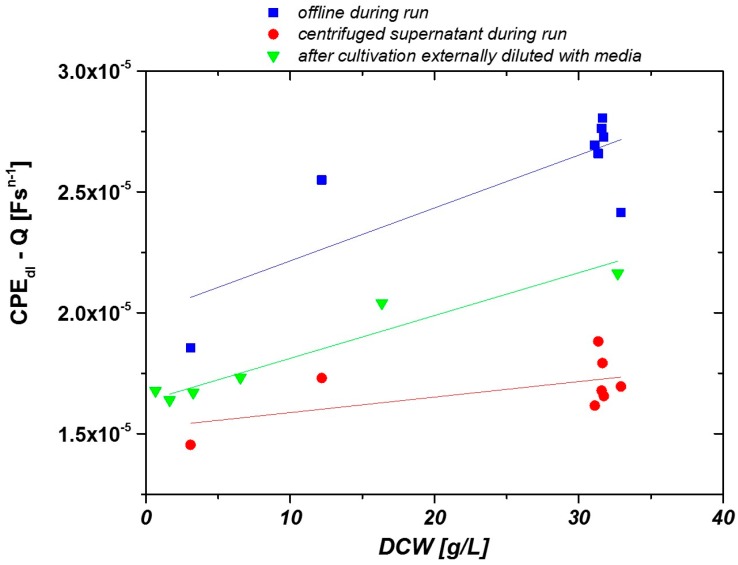
Offline measurements of clarified fermentation supernatant (**red circles**), cultivation broth (**blue squares**) and harvested cells resuspended in buffer at different concentrations (**green triangles**).

The resulting double layer capacitances for the centrifuged cultivation supernatants (circles), for cultivation broth samples (squares), and for resuspended cells (triangles) are shown in Figure 3 as a function of DCW. FCM measurements of all samples within this study were performed to verify the viability of the cells. As less than 5% percent of the cells were dead throughout all samples, DCW and VVC are equivalent.

The composition of the cultivation supernatant changes as a function of time due to different cell concentrations. The impedance signal of the clarified fermentation supernatant is plotted as a function of the biomass concentration of the samples before centrifugation in Figure 3 (red circles). The slope of the curve is very low (k = 6.4 × 10^−8^), indicating a low impact of the medium on the overall impedance signal.

Resuspending centrifuged biomass in cultivation media at different concentrations resembles a significant linear fit between the biomass concentration (k = 1.8 × 10^−7^) and impedance signal (Figure 3, green triangles). While cultivation samples with cells were measured in the offline mode (blue squares), the fit of the calibration curve was not as nice as for the calibration made from diluted samples. We believe that this noise is a result of different storage times between sample taking and measuring the samples, as storing of the samples can have a severe impact on the morphology of the cells and thus on the impedance signal [36]. Furthermore, temperature control was not possible and sedimentation of the cells might have impacted the measurements. Therefore, we decided to construct a prototype with temperature control and a constant flow-through as described above and measured samples in the online mode.

### 3.3. Impedance Measurement in Online Mode Using the Developed Prototype

To remove noise in the impedance, the signal due to storage of the samples between sample taking and measurements, the different temperatures during analysis and the sedimentation of the cells, the developed prototype is installed in the online mode as shown in Figure 1.

**Figure 4:**
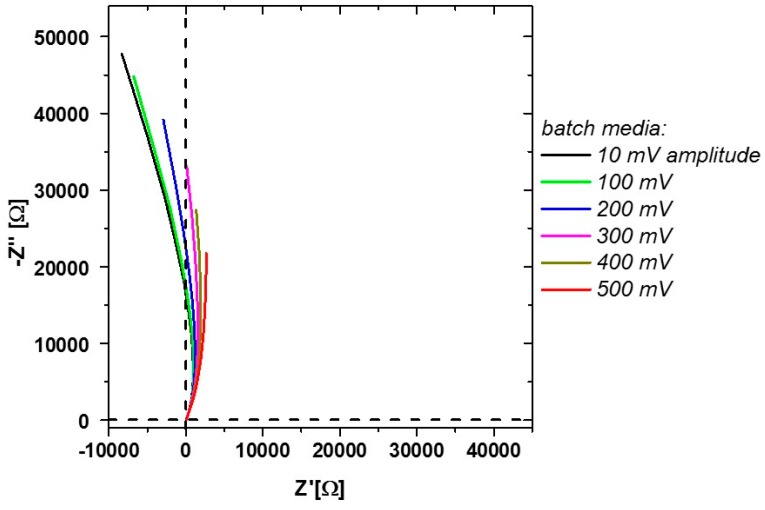
Measurement during flow-through including changes in the measured media sample. Higher amplitudes shift the differential resistance to positive values.

Deviations of the ideal impedance signal compared to offline measurements can be found in flow-through mode. Measurements at 100 to 300 mV amplitude of the media without cells showed loops to negative differential resistances at lower frequencies (Figure 4). This indicates a slightly changing double layer resistance R_dl_ over the observed recording time of one impedance spectrum, respectively.

Since those impedance responses can already be spotted before inoculation, it might be related to a charge transfer reaction of media components on the electrode. An electrolyte-related change in the stability of the electrode may lead to passivation reactions. These can result in those negative differential resistances (changes of the current in respect to the applied voltage) [37]. Furthermore, negative resistances can occur during absorption/reactions on the electrode [38]. Similar impedance responses are obtained, for example for glucose, with Ni-containing electrodes [39]. Since the used stainless steel electrodes contain Ni as well, such reactions may also be possible in this case. However, since such negative resistances are not found during offline measurements of sugars, flow-through during the measurements seems to have a major effect on the spectra. Rather high amplitudes of 500 mV do not show the pronounced behavior seen at lower amplitudes and are therefore used for the later measurements in flow-through mode (Figure 4).

**Figure 5:**
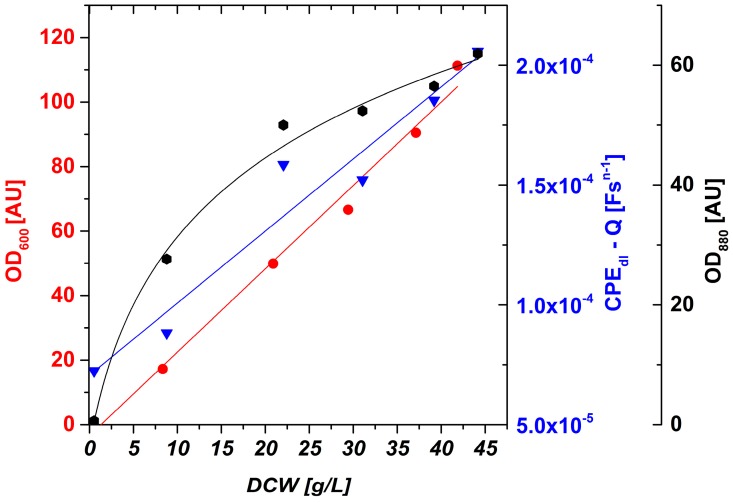
OD_600_ offline, OD_880_ inline and impedance online (CPE_dl_-Q) signals as a function of DCW. OD_600_ online and CPE_dl_-Q online show a linear behavior and were thus fitted by a linear regression. OD_880_ inline shows saturation at higher DCW and was thus fitted by a logarithmic curve.

Furthermore, DCW, OD_600_ offline and OD_880_ inline were monitored. To show linearity between DCW and OD_600_ offline, and OD_880_ inline and the impedance signal, the correlation between those signals is plotted in Figure 5 using OriginLab software (OriginLab Corporation, Northampton, MA, USA). In general, media contributions are negligible with increasing electrode distance α during these measurements. Hence, values for the double layer capacitance could also be determined even with simplified fitting routines using a resistance for the real axis offset and a CPE element for the double layer capacitance when R_dl_ is far too high, especially in the beginning of the cultivations.

OD_880_ inline shows a linear behavior up to about 20 g/L DCW. At higher biomass concentrations, saturation effects according to Lambert-Beer’s law occur and result in a nonlinear fit with low sensitivity. OD_600_ offline shows a good linear fit also at high DCW, but samples have to be taken and processed (diluted) and the signal is not available in the online mode. The CPE_dl_-Q online signal shows a high linearity (R^2^ = 0.94) in a very dynamic biomass concentration range.

**Figure 6:**
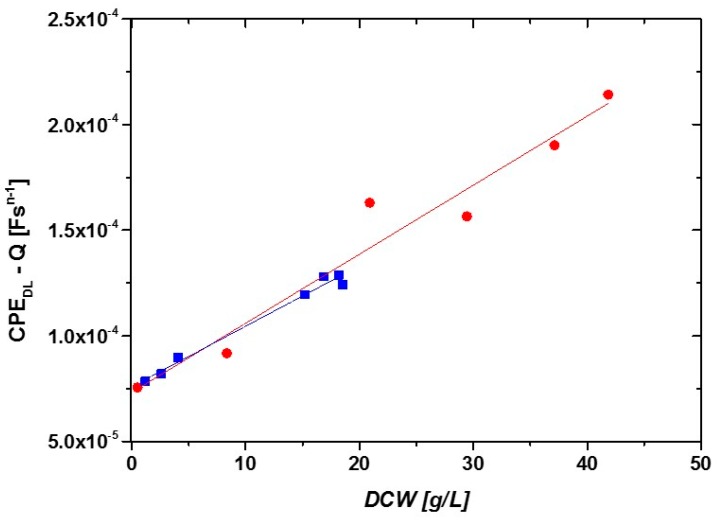
Correlation between DCW and impedance signal (CPE_dl_-Q) measured in online mode during two fed batch cultivations with different feeding strategies.

To show the reproducibility of the developed measurement principle, a correlation between DCW and CPE_dl_-Q was established for two different cultivations (Figure 6).

**Figure 7:**
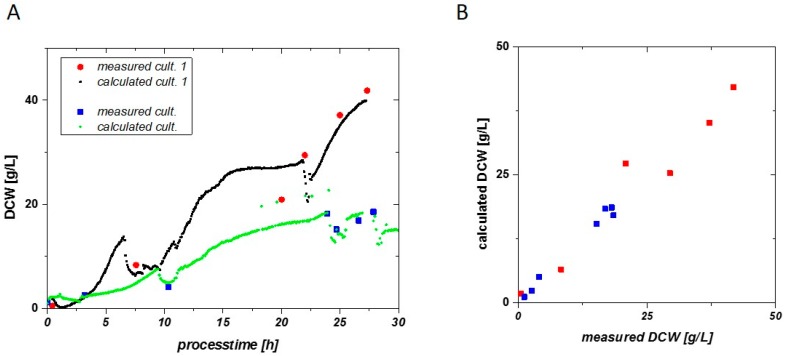
(**A**) DCW can be calculated by the online impedance signal very accurately. Determination of the VCC via double layer capacitance is reproducible for different *E. coli* cultivations; (**B**) Calculated DCW vs. measured DCW.

Both fits show very similar slopes (Figure 6) and a high R^2^ (0.94 and 0.98). The correlation between DCW and CPE_dl_-Q was used to calculate the DCW using the impedance signal for two different cultivations exhibiting different specific growth rates, µ. Figure 7A shows the measured and the calculated DCW as a function of time for the two different cultivations and Figure 7B shows the correlation between calculated and measured DCW.

Very good reproducibility is found in two cultivations, even with very different specific growth rates applied. The quality for process control strategies can further be highlighted in Figure 7B. The calculated DCW vs. the measured DCW is situated along the first median. Values not situated along the first median indicate for the overall error in the fitting routine, compared to a residual analysis. Only measurements in the late fed batch phase of cultivation 1 are way off the first median and result in an overestimation of biomass at this time point.

## 4. Conclusions

Within this study we presented the feasibility of measuring DCW of *E. coli* using impedance spectroscopy at low frequencies. The proposed technique is easily applicable and has a high dynamic range from low cell densities at the beginning of the batch phase to cell densities beyond 40 g/L DCW. It is shown that this technique can be applied in an industrial fermentation strategy with high resolution and high reproducibility. Furthermore, the developed prototype can be easily rebuilt, as standardized parts were used. We believe that this measurement technique will greatly facilitate bioprocess development as VCC can be measured in real time at low but also at high cell densities with low background interferences.

## Figures and Tables

**Table 1:** Fitting results of CNLS fit given in Figure 2B with corresponding error estimations (Chi squared: 1.1825 × 10^−3^; Sum of squares: 0.1596) and calculated real capacitance according to Equation (3).

	L_offset_ [H]	R_offset_ [Ω]	R_media_ [Ω]	C_media_ [F]	R_dl_ [Ω]	CPE_dl_-Q [Fs^n−1^]	CDP_dl_-n [-]	C_dl_ [F]
**abs. value for 25 g/L**	2.18 × 10^−6^	218.6	78.6	1.66 × 10^−5^	3.95 × 10^5^	2.14 × 10^−5^	0.82	3.42 × 10^−5^
**NRMSE [%] for 25 g/L**	10.70	0.19	5.10	4.24	4.85	0.42	0.19	-
**abs. value for 1 g/L**	1.83 × 10^−6^	139.6	83.69	1.15 × 10^−5^	4.44 ×10^6^	1.62 × 10^−5^	0.87	3.07 × 10^−5^
**NRMSE [%] for 1 g/L**	8.48	0.20	3.31	2.67	38.23	0.38	0.15	-

## Abbreviations

VCC—viable cell concentration, FCM—flow cytometry, AC—alternating current, OD—optical density, DCW—dry cell weight, C—capacitance, CPE_dl_—constant phase element of the double layer, R_dl_—double layer resistance, R_offset_—offset resistance, L_setup_—inductivity of the measurement setup, Z—impedance, ω—arc frequency, NRMSE—root-mean-square-deviation.
